# CD19^+^ B cell depletion: a novel strategy to alleviate ischemic stroke damage

**DOI:** 10.3389/fimmu.2025.1528471

**Published:** 2025-04-17

**Authors:** Yu Xu, Jing Peng, Yizhong Yan, Min Gao, HongJing Zang, Lamei Cheng, Yu Zhou

**Affiliations:** ^1^ Department of Neurosurgery, The Second Xiangya Hospital of Central South University, Changsha, China; ^2^ Institute of Reproductive and Stem Cell Engineering, School of Basic Medical Science, Central South University, Changsha, China; ^3^ National Engineering Research Center of Human Stem Cell, Changsha, China; ^4^ Department of Radiology, The Second Xiangya Hospital of Central South University, Changsha, China; ^5^ Department of Pathology, The Second Xiangya Hospital of Central South University, Changsha, China; ^6^ Hunan Guangxiu Hi-tech Life Technology Co. Ltd, Changsha, China

**Keywords:** ischemic stroke, B cells, anti-CD19 antibody, inflammation, meningeal immunity

## Abstract

**Background:**

Ischemic stroke, accounting for approximately 80% of all stroke cases, is a major public health challenge and a leading cause of death and disability worldwide. Current treatments primarily involve thrombolytic therapy, limited to a 4.5-hour window due to the risk of complications, underscoring the need for new therapeutic targets. Systemic inflammation plays a critical role in stroke progression, with immune cells infiltrating the brain and exacerbating damage. B cells, in particular, have been implicated in stroke pathogenesis, although their exact role remains contentious. This study examines anti-CD19 antibody (aCD19 Ab) treatment in a stroke model to determine if CD19^+^ B cell depletion can reduce infarct size and alleviate inflammation.

**Results:**

This study investigated whether temporary inhibition of B-cell activity using an aCD19 Ab could alleviate ischemic brain injury in a stroke mouse model by regulating cerebral and systemic immune reactions. Mice subjected to middle cerebral artery occlusion (MCAO) exhibited significant reductions in infarct size and brain edema, prolonged post-MCAO survival, and improved behavioral outcomes following aCD19 Ab treatment. Transmission electron microscopy (TEM) and Computed Tomography Angiography (CTA) results revealed a reduction in microvascular endothelial edema, decreased mitochondrial damage in neurons, reduced neuronal apoptosis, and a favorable reconstruction of the cerebral vascular network. Additionally, B cell inhibition reduced pro-inflammatory cytokines and immune cells in the brain and peripheral circulation. The immune response alterations observed in the MCAO/R group were consistent with the trends indicated by stroke patient data.

**Conclusions:**

Temporary inhibition of B-cell activity via aCD19 antibody injection alleviated ischemic brain injury in a mouse model of stroke by suppressing systemic immune reactions. Changes in immune cells within the meninges may play a role, and further investigation is needed to understand the mechanisms involved. These findings suggest that cerebral and systemic immune responses contribute to the pathogenesis of ischemic stroke, and temporary B cell depletion may represent a potential therapeutic target for stroke therapy.

## 1 Introduction

Ischemic stroke, accounting for approximately 80% of all stroke cases, remains a major challenge in public health and a leading cause of death and long-term disability worldwide (1). The burden of this disease is particularly profound in low- and middle-income countries, presenting a significant economic impact due to the long-term care required for stroke survivors (2). Stroke treatment represents a formidable global healthcare challenge, primarily relying on thrombolytic therapy within specific time windows. Beyond the therapeutic window of 4.5 h, the benefits of tPA are outweighed by its risks, with the incidence of hemorrhagic transformation increasing dramatically (3). Limited therapeutic options highlight the urgency to identify novel therapeutic targets for ischemic stroke (4). As systemic inflammation is strongly linked to the occurrence and progression of stroke (5, 6), researchers have focused on the role of both the central and peripheral immune systems in understanding stroke development and exploring potential therapeutic approaches (7). Inflammation can exacerbate damage during and after an ischemic stroke, triggering multiple damage reactions (6). This has led to a significant interest in immunomodulatory treatments as potential interventions to mitigate these harmful effects.

When a stroke occurs, peripheral immune cells including monocytes/macrophages, neutrophils and lymphocytes invade the brain, initiating further inflammatory reactions (6). This response can exacerbate the damage caused by the stroke itself. Analysis of peripheral blood from stroke patients often shows elevated levels of these immune cells and associated cytokines, particularly in those with worse symptoms and prognoses (8). This complex immune response illustrates the significant role of the immune system in the processes associated with stroke, leading researchers to explore the potential involvement of immune cells, particularly B cells, in the pathogenesis of ischemic strokes (9). The role of B cells in stroke pathogenesis remains a contentious topic within medical research, with studies presenting conflicting evidence (10–12). Some research suggests that B cells have minimal impact on stroke outcomes compared to other immune cells, indicating that their presence or suppression does not significantly alter the course of recovery or damage (13, 14). On the other hand, other studies advocate for a beneficial role of B cells, particularly highlighting their potential in contributing to long-term neural function restoration (10, 11, 15). This discrepancy points to the need for further research to clarify the roles of different immune cells, including B cells, in stroke pathophysiology and recovery.

Moreover, B cells have been observed to accumulate in the meninges following a stroke, underscoring the significant role of the meningeal lymphatic system in the post-stroke immune response (16). These B cells interact with other immune cells, contributing to CNS inflammation and tissue damage (17, 18). Meningeal lymphatic vessels (MLVs) act as a physical conduit for antigens and immune cells from the central nervous system to enter the peripheral lymph nodes (16). This facilitates the activation of systemic immune response, allowing immune cells to traverse a compromised blood-brain barrier (BBB) to the brain (19).

CD19 and CD20 are predominantly B cell-restricted surface markers, providing an opportunity for selective B cell-targeted immunotherapy. CD19, a critical surface protein on B cells, plays a pivotal role in regulating B cell-mediated immune responses. Due to its function in modulating B cell activity, CD19 has emerged as a prominent therapeutic target in the treatment of autoimmune diseases and cancers (20, 21). CD19 is expressed throughout B cell development, from pro-B cells to pre-B cells, and persists in some plasmablasts (PBs) and plasma cells (PCs). In contrast, CD20 is expressed in most B cell stages except for pro-B cells, PBs, and PCs. Therefore, antibodies targeting CD19 may have broader effects on the B cell compartment compared to anti-CD20 antibodies (22–24).

Beyond their role in antibody production, B cells—particularly PBs and PCs—contribute to cytokine production and immune modulation. Our previous studies demonstrated that aCD19 Ab treatment improved ovarian pathology and function in polycystic ovary syndrome (PCOS) mice by alleviating local and systemic inflammation (25).

B-cell depletion therapies, particularly those utilizing anti-CD20 antibodies such as rituximab, have been associated with prolonged immunosuppression and an increased risk of infections, making them less viable for some autoimmune disease treatments (23, 26). This study investigates the efficacy of CD19^+^ B cell depletion using an anti-CD19 (aCD19) antibody in the Middle Cerebral Artery Occlusion (MCAO) model, a well-established animal model of ischemic stroke. The objective is to identify an effective B cell inhibition strategy that minimizes prolonged immunosuppression and reduces associated infection risks (23).

By assessing whether temporary B cell inhibition during the acute phase of stroke can decrease infarct size and mitigate harmful inflammatory responses, the research aims to elucidate the role of B cells in ischemic stroke and evaluate their potential as therapeutic targets. Insights from this study may guide the development of interventions to protect the ischemic penumbra from further damage post-stroke and inform preventive strategies for patients at high risk of stroke.

## 2 Methods and materials

### 2.1 Animal and MCAO model

**Figure 1:**
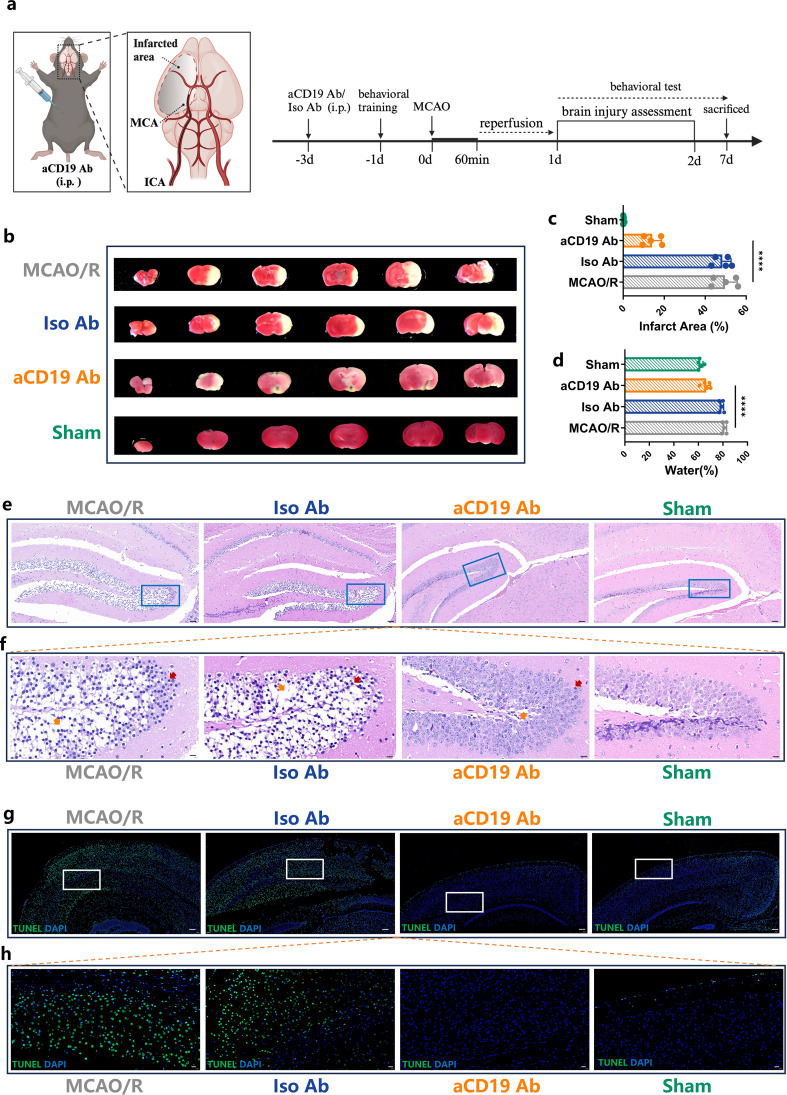
aCD19 Ab treatment reduced the infarct size, inhibited apoptosis and alleviated edema in MCAO/R models. **(a)** Mice were intraperitoneally injected with aCD19 Ab (10 mg/kg) or Iso Ab (10 mg/kg) 3 days before 1 hour of tMCAO, after which reperfusion was allowed, and outcomes were assessed for 7 days. Created via BioRender.com. **(b)** TTC staining displayed the infarct area of different treatments. The white area showed the infarct area. **(c)** Statistical analysis of the ratio of the infarct area to the total brain volume. Data were presented as the percentage of white infarct area. Data were presented as mean ± SD (n=5). ****p < 0.0001. **(d)** Statistical analysis of the ratio of the water content of the brain. Data were presented as the relative percentage of water content. Data were presented as mean ± SD (n=5). ****p < 0.0001. **(e)** H&E staining results showed the ischemic area in 40 times magnification (Scale bar=50 μm). **(f)** The rectangular region of the upper image in each group was magnified and placed in the lower panel at 100 times amplification (Scale bar=10 μm). Red arrows point at the nuclear pycnosis; Orange arrows point at the formation of vacuoles after necrosis. **(g)** TUNEL staining results exhibited the apoptotic cells in the cerebral tissue at 20 times amplification (Scale bar=100 μm). **(h)** TUNEL staining: The rectangular region of the upper image in each group was magnified and placed in the lower panel at 60 times amplification (Scale bar=20 μm).

All animal procedures were approved by the Animal Care and Use Committee of The Second Xiangya Hospital of Central South University and performed in line with the National Institutes of Health Guide for the Care and Use of Laboratory Animals. Male C57BL/6 mice (8-10 weeks old, 20-25 g, Hunan Kingda Laboratory Animal Co., China) were kept in a pathogen-free environment with consistent temperature and humidity, 12-hour light and dark cycles, and free access to a standard diet and water. As shown in **Figure 1a**, mice were randomly assigned to receive either monoclonal rat anti-mouse CD19 antibody (1D3, Bio X Cell) (10 mg/kg) or Isotype control antibody (2A3, Bio X Cell) (10 mg/kg) by intraperitoneal injection 3 days in advance, creating the CD19 Ab-treatment group and the Iso Ab-treatment group, respectively. The Sham and MCAO/R group received an equivalent volume of Dulbecco’s Phosphate-Buffered Saline (DPBS).CD19^+^ B cell depletion was confirmed by flow cytometry (**Supplementary Figure S1a**).

Focal cerebral ischemia was induced using the transient middle cerebral artery occlusion (tMCAO) method. Mice were anesthetized with 1–2% isoflurane, and a 6-0 monofilament nylon suture with a rounded tip was inserted into the internal carotid artery to occlude the middle cerebral artery for 60 minutes. Reperfusion was initiated by gently withdrawing the suture (27). Sham-operated mice underwent the same surgical procedure but without middle cerebral artery occlusion. Throughout the procedure, rectal temperature was maintained at 37°C using a heating pad to ensure physiological stability. Following recovery from anesthesia, neurological deficits were assessed to confirm the successful induction of ischemic stroke, as determined by the Zea Longa score (Section 2.6.1). Peripheral blood was collected for flow cytometry analysis, and fresh mouse brain tissues were harvested at 24 and 48 hours post-tMCAO for 2,3,5-triphenyltetrazolium chloride (TTC) staining and dry-wet ratio evaluation. For histological and immunohistochemical analyses, mice were sacrificed 24 hours after tMCAO. The brains were harvested and fixed in 4% paraformaldehyde (PFA) overnight before being processed for paraffin embedding.

### 2.2 Evaluation of infarct area

Infarct size was measured by 2,3,5- TTC (Sigma-Aldrich, USA) staining as described earlier (28). Briefly, the brains were rapidly removed and sliced into six 2-mm sections and incubated in 2% TTC solution at 37°C for 15 minutes. The infarct area was measured using Image J software (NIH, Bethesda, MD, USA).

### 2.3 Cerebral edema

The severity of cerebral edema was assessed 48 hours post-modeling using the wet-dry weight method to determine brain moisture content. The brain was promptly dissected into left and right hemispheres, and each hemisphere was weighed to obtain its wet weight. Subsequently, the tissues were dried at 105°C overnight and reweighed to determine their dry weight. The percentage of water content was then calculated using the following formula: [(wet weight - dry weight)/wet weight] ×100%. This method provides a quantitative measure of cerebral edema, allowing for an objective evaluation of post-ischemic brain swelling.

### 2.4 Histopathological examination

Brain tissues were embedded in paraffin and sectioned at 3.0-3.5μm thickness. The sections were dewaxed and stained with hematoxylin and eosin (H&E) for microscopic evaluation.

For immunofluorescence (IF) analysis of brain tissue, the sections were blocked with 10% normal goat serum (Solarbio, China) and 0.03% Triton X-100 in PBS for 15min, followed by overnight incubation at 4°C with the following primary antibodies: mouse anti-GFAP (CST, 3670S, 1:200), rabbit anti-Ang 1 (Proteintech, 23302-1-AP, 1:200), rat anti-LYVE-1 (R&D, MAB2125, 1:100), rabbit anti-CD31 (Proteintech,28083-1-AP, 1:100), rabbit anti-CD19 (Abcam, ab227019, 1:600), and rabbit anti-MPO (Abcam, ab9535, 1:100). The sections were subsequently incubated with fluorescent secondary antibodies. Nuclei were counterstained with DAPI (Sigma-Aldrich, USA), and positive cells were visualized using a laser scanning confocal microscope (Olympus, Japan). ImageJ software was used for quantification.

For immunohistochemical (IHC) analysis, tissue sections were rehydrated, blocked, and incubated overnight at 4°C with primary antibodies (Abcam), including anti-CD19 (1:100, ab227019), anti-CD3 (1:10, ab135372), and anti-myeloperoxidase (MPO) (1:100, ab208670). The sections were then treated with a secondary antibody (1:500, ab6127) at room temperature. Diaminobenzidine (DAB) (ZLI-9018, ZSGB-BIO, China) was used for color development, followed by hematoxylin counterstaining. The integrated optical density (IOD) values were quantified using ImageJ software.

### 2.5 TUNEL histochemistry.

Paraffin-embedded brain sections were fixed in 4% paraformaldehyde and subsequently dewaxed. Apoptotic cell detection was performed using the One-Step TUNEL Apoptosis Assay Kit (Beyotime, China) according to the manufacturer’s protocol. The slides were then examined under a fluorescence microscope for visualization of apoptotic cells.

### 2.6 Neurological scoring and behavioral assessment

Behavioral assessments were conducted within 7 days after MCAO/R to evaluate locomotor activity, grip strength, somatosensory motor function, and short-term memory.

#### 2.6.1 Zea Longa score

Neurological impairment was assessed using the Zea Longa scoring system, which categorizes deficits as follows: 0 (Normal movement), 1 (Incomplete extension of the contralateral forelimb when lifted by the tail), 2 (Circling or twisting toward the contralateral (paralyzed) side when walking), 3 (Spontaneous falling to the contralateral side or falling when nudged), and 4 (Absence of spontaneous movement or loss of consciousness) (29).

#### 2.6.2 Grip strength test

Grip strength was assessed using a grid-connected electronic grip strength meter (Bioseb, France). Mice were allowed to grasp the metal grid while the tail was gently pulled in the opposite direction. The peak force generated before the animal released the grip was recorded. Each mouse underwent three trials, and the mean grip strength was expressed in kg.

#### 2.6.3 Tape removal test

Mice were habituated to a testing container before the experiment. Small circular adhesive stickers (5 mm in diameter) were attached to the bilateral forepaws using fine forceps and gently pressed to ensure adherence. Each mouse was then placed in a testing beaker for 150 seconds. The removal time for each tape was recorded via video analysis. If a mouse successfully removed both stickers within the time frame, the precise removal time was noted. If a mouse failed to remove one or both stickers, the trial was terminated at 150 seconds, with the removal time recorded as 150 seconds. Each mouse underwent three trials, with an inter-trial resting period of approximately 30 minutes.

#### 2.6.4 Open Field Test

The Open Field Test was conducted to assess hyperactivity and anxiety-related behavior by evaluating locomotor activity. The testing arena was a black plexiglass square enclosure (60 cm × 60 cm × 25 cm), with a designated center region (30 cm × 30 cm) demarcated using Vinyl Electrical Tape (Tartan 1710). Mice were placed in the enclosure for 5 minutes while their movements were recorded. Behavioral parameters, including total distance traveled and time spent in the center, were quantified through video analysis. This test is based on the principle that mice instinctively prefer the security of peripheral areas over exposed central regions, providing insights into their anxiety levels.

### 2.7 MRI scanning

*In vivo* imaging was conducted on Day 2 using a 3.0 Tesla NMR spectrometer (Bruker Biospin, Billerica, MA) equipped with a Micro 2.5 gradient system (with a maximum gradient strength of 100 G/cm), using a manufacturer-provided animal imaging probe. A 20 mm diameter volume coil functioned as both the radiofrequency transmitter and receiver. Image acquisition utilized a three-dimensional (3D) T2-weighted fast spin echo sequence (FSE) with the following parameters: echo time (TE)/repetition time (TR) = 40/700 ms, 16 mm x 16 mm x 16 mm field of view, and 128 x 128 x 80 matrix (resulting in a resolution of 0.125 mm × 0.125 mm × 0.2 mm), echo train length = 4, number of averages = 2, flip angle = 40°, and bandwidth (BW) = 100 kHz. The total imaging time spanned approximately 30 minutes. Subsequently, the T2-weighted FSE images were resampled to achieve an isotropic resolution of 125 μm.

### 2.8 Micro-CT scanning.

MICROFIL^®^ compounds were utilized for visualizing the microvasculature. Injected under physiological pressure, these compounds filled and rendered the microvasculature opaque, forming a three-dimensional cast after solidification. A sine wave perfusion pump connected to the arterial cannula facilitated perfusion, with the right atrium serving as a drainage vent. Saline perfusion removed visceral blood volume. Post-perfusion, MV-130 Red was infused via the aortic cannula, and refrigeration at 4°C overnight aided polymerization. The brains were harvested on the following day.

Ex vivo perfused mouse brains using MICROFIL compounds underwent micro-CT scanning (Inveon, Siemens Inc., Knoxville, TN, USA) with settings including 85 kV voltage, 200 µA output current, 3 µm spatial resolution, 0.2° rotation step through 360° (yielding 1801 projections), and a 1 mm Aluminum filter, along with 8-frame averaging. Each brain’s scanning process took approximately 1 hour. Cobra software (version 6.3.39.0, EXXIM Computing Corporation, Pleasanton, CA, USA) facilitated image reconstruction. While standardized settings were applied, individual fine-tuning enhanced reconstruction quality beyond normalized automatic deconvolution. Fine-tuning parameters included a smoothing value of 0, misalignment compensation between 52-60, ring artifacts reduction between 4-6, and beam-hardening correction between 20-30%. Subsequently, data were imported into Radiant DICOM Viewer (Poland).

### 2.9 Transmission electron microscopy (TEM) scanning

Immediately after the mice were anesthetized and perfused, the brain was cut into 50 μm thick transverse sections using a vibratome in ice-cooled PBS. The sections were then immersed in 1% osmium for 30 minutes at room temperature, rinsed three times for 10 minutes each in PBS, and dehydrated through immersions in ascending concentrations of ethanol followed by three times in propylene oxide. The sections were removed from propylene oxide and immersed in Durcupan resin. The next day, an ACLAR embedding film was coated with a thin layer of resin. The resin was polymerized and square areas of interest (about 2x2 mm) were selected, glued onto the tip of resin blocks with Superglue, and cured in the 55°C oven for 1 hour. A few semi-thin sections (0.5-1 μm thick) were cut. The sections were transferred to a SuperFrost slide using a perfect loop and dried by placing the slide on a heating plate. Silver to silver-gold ultrathin sections (60-80 nm thick) were cut, collected on copper mesh grids using fine inverted tweezers, dried on a filter paper, and transferred to a drop of lead citrate for 2 minutes. The grids were rinsed in three successive baths of double distilled water and dried on a filter paper before being stored in a grid box for electron microscopic examination.

### 2.10 Flow cytometry

Peripheral blood was collected from the orbital venous plexus, and the erythrocytes were lysed. Cells were centrifuged for 5 min at 350 RCF and resuspended in 100 μl FACS buffer (BD Biosciences, USA). Leukocyte single-cell suspensions were then incubated with a selected fluorophore-conjugated antibodies as: Per-CP anti-mouse CD45 (clone 30-F11), FITC anti-mouse CD3 (clone 17A2), APC anti-mouse CD4 (clone GK1.5), PE anti-mouse CD8a (clone 53-6.7), APC anti-mouse CD19 (clone 6D5), APC anti-mouse LY6G (clone 1A8), PE anti-mouse CD11b(clone M1/70), which were purchased from Biolegend (San Diego, CA, USA). Antibodies used in the human Peripheral blood are listed as: Pecy5.5 anti-human CD45 (clone HI30), FITC anti-human CD3 (clone OKT3), PE anti-human CD4 (clone OKT4), APC anti-human CD8 (clone SKI), PE anti-human CD19 (clone HIB19).

The mouse brain was rapidly removed after left ventricular perfusion and placed in pre-cooled, complete Roswell Park Memorial Institute (RPMI) 1640 (Gibico, Waltham, MA, USA) medium. After mechanical dissociation, it was digested in collagenase/dispase (1 mg/mL) and DNAse (10 mg/mL; both from Sigma-Aldrich, St Louis, MO, USA) at 37°C for 1 hour and then neutralized. The cell suspension was filtered through a 70 μm filter. Leukocytes were harvested from the interphase of a 70%/30% Percoll (Sigma-Aldrich, St Louis, MO, USA) gradient. Cells were washed and blocked with anti-mouse CD16/32 (clone 93), and then stained with fluorophore-conjugated primary antibodies: FITC anti-mouse CD3 (clone 17A2), PE anti-mouse CD11b (clone M1/70), PerCP-Cy5.5 anti-mouse CD45 (clone 30-F11), and APC anti-mouse CD19 (clone 6D5). All antibodies were commercially purchased from Biolegend (San Diego, CA, USA). Fluorescence data were collected with a Accuri C6 cell sorter (BD Biosciences, USA). Further data analysis and illustration were analyzed with FlowJo software.

### 2.11 Real-time PCR

Mice brain tissue total RNAwas extracted by Trizol (Sigma-Aldrich, St Louis, MO, USA). cDNA was prepared through reverse-transcription using a RevertAid RT kit (Fermentas, Lithuania). Real-time PCR was performed using a SYBR Green PCR mix on an ABI Prism 7500 (Applied Biosystems, USA). Relative expression levels were normalized using GAPDH as a control and gene expression was subsequently quantified using the 2^-ΔΔCt^ method. The sequences of PCR primers are as follows: *Gapdh*, Forward 5 ‘-GGTGTCTCCTGCGACTTCA-3’, Reverse 5 ‘-TAGGCCTCTTGCTCAGT-3’; *Il-2*, Forward 5 ‘-GTGCTCCTGTCAACAGCG-3’, Rreverse 5 ‘-GGGGGGAGTTTTCAGTCCTGTA-3’; *Il-6*, Forward 5 ‘-CACTCACAAGTCGGAGGCT-3’, Reverse 5 ‘-CTGCATGCATCATCGTGTGT-3’; *Il-10*, Forward 5 ‘-TGAGGCTGTCATCGATTT-3’, Reverse 5 ‘-TGGCCTGTAGTACACCTGTGG-3’; *Tnf-α*, Forward 5 ′ -ATGAAGTCATCAGC-3′, Reverse 5′-CTCCCACTGTGTGTGTA-TTA-3′.

### 2.12 Human samples

Five ischemic stroke patients and one brain trauma patient were recruited from the Neurosurgery Intensive Care Units of the Second Xiangya Hospital, between June 2018 and June 2019. Written informed consent was obtained from all patients or their legally authorized representatives before inclusion. The study was approved by the Institutional Review Board of the Second XiangYa Hospital of Central South University. Eligibility required a confirmed ischemic stroke diagnosis through neuroimaging. Exclusion criteria included evidence of hemorrhagic stroke, absence of ischemic stroke, or indeterminate ischemic stroke diagnosis. Administration of tPA and thrombectomy were not exclusionary. Neurological function and disability were assessed using the Glasgow Coma Scale (GCS), the Modified Rankin Scale (mRS), the National Institutes of Health Stroke Scale (NIHSS), and the Barthel Index (MBI) (30). Fasting venous blood was collected into acid-citrate-dextrose (3.2%) sterile tubes to examine immune cell changes, and cell-free plasma was isolated for subsequent ELISA. This decompressive craniectomy surgery requires opening the dura mater to perform duraplasty, which relieves high intracranial pressure caused by swollen brain tissue. In patients with open cranial trauma, portions of the dura mater in the damaged region are often trimmed and removed during surgical debridement. These dura mater samples, which would otherwise be discarded as medical waste, were used as our study samples. This approach did not interfere with the surgical procedure or treatment outcome, and it did not cause additional harm to the patients. Regarding the sampling methodology, we recognize the importance of defining the number of ROIs and the total surface area sampled. However, due to ethical constraints and the limited availability of human meningeal tissue, it was not feasible to sample multiple ROIs or collect a large enough meningeal tissue sample to allow for multiple independent regions of analysis. The meningeal tissue obtained during surgery is typically small (~1 cm × 0.5 cm), particularly in patients undergoing decompressive craniectomy for stroke. Obtaining a larger tissue sample would compromise dural integrity and water-tight closure, which is crucial for patient recovery. Meningeal samples were obtained through surgical extraction of a small dura mater section from the affected cerebral hemisphere and stained. Each patient sample was analyzed by experienced pathologists, who directly selected 3 high-power fields (40×) for quantification. Clinical characteristics are summarized in **Table 1**.

**Table 1:** Clinical and laboratory characteristics of the stroke patients.

	Reference Values	Patient 1	Patient 2	Patient 3	Patient 4	Patient 5
**Sex**	M/F	M	F	M	F	F
**Age**	/	36	47	55	62	64
**GCS**	/	13	12	11	10	10
**mRS**	/	3	4	4	5	5
**NIHSS**	/	18	20	22	33	36
**MBI**	/	62	45	43	36	18
**CD19^+^ B cells**	5~22%	18	17	20	20	28
**Neutrophils**	40.0~75.0%	87.7	86.5	89.4	90.3	92.9
**NEUT# in PB**	1.8-6.3 10^9^/L	11.5	10.9	12.4	13.7	14.5
**CD3^+^CD4^+^ T cells**	33~58%	10.92	12.63	9.48	76	19
**CD3^+^CD8^+^ T cells**	13~39%	9.43	9.18	41.57	89	4
**IL-2**	0.00~5.71 pg/ml	10.37	12.94	64.73	80	6
**IL-6**	0.00~5.30 pg/ml	14.52	46.83	78.52	62	22
**IL-10**	0.00~4.91 pg/ml	13.76	45.97	71.69	59	28
**TNF-α**	0.00~2.31 pg/ml	12.36	59.13	77.46	68	19
**CRP**	0-6.00 mg/L	65.6	41.6	76.9	71.7	98.6
**Length of stay**	/	13	20	41	62	10 (death)

GCS, Glasgow Coma Scale; mRS, Modified Rankin Scale; NIHSS, The National Institutes of Health Stroke Scale; MBI, Modified Barthel Index; CRP, Plasma C-reactive Protein; NEUT#, the number of neutrophils in peripheral blood.

### 2.13 Statistical analysis

Statistical analyses were performed using GraphPad Prism 9.4 software. Data are presented as mean ± standard deviation (SD). Student’s t-test or one-way analysis of variance (ANOVA) with Tukey’s *post-hoc* test was used for statistical comparisons, as appropriate. A p-value of less than 0.05 was considered statistically significant.

## 3 Results

### 3.1 aCD19 Ab treatment reduced infarct volume, inhibited apoptosis, and alleviated edema in MCAO models

To investigate the role of anti-CD19 antibody (aCD19 Ab) in the pathophysiological process of ischemic stroke, mice were preconditioned with intraperitoneal aCD19 Ab injection. The efficacy of aCD19 Ab in depleting peripheral blood (PB) B cells was first confirmed (at rate of 99.1%), as shown in **Supplementary Figure S1a**. Additionally, baseline B-cell levels in untreated mice, as well as in the Iso Ab and aCD19 Ab groups before MCAO induction, were quantified using flow cytometry (FC) (**Supplementary Figure S1b**).

To assess the therapeutic effects of aCD19 Ab on ischemic brain injury, TTC staining was performed to measure infarct volume following middle cerebral artery occlusion/reperfusion (MCAO/R). The MCAO/R group exhibited a stable infarction size, with an infarct volume of approximately 49.6%, as illustrated in **Figures 1b, c**. Notably, aCD19 Ab treatment significantly reduced infarct volume to approximately 14.1%, a reduction to about one-fifth of that observed in the MCAO/R and Iso Ab groups.

In the acute phase of stroke, intracranial hypertension due to cerebral edema is a major contributor to patient mortality, often leading to life-threatening brain herniation. To evaluate this, we measured brain water content, an indicator of cerebral edema. Compared to the Sham group, the infarcted hemisphere in the MCAO/R group exhibited a marked increase in brain water content, rising from approximately 61.6% to 81.2%. However, aCD19 Ab treatment significantly mitigated this edema, reducing water content to approximately 66.4%, a value close to that of the Sham group (**Figure 1d**).

To further investigate the effect of aCD19 Ab on tissue integrity following MCAO/R, we assessed two key neuronal populations in the infarcted area: cortical and hippocampal neurons, which play critical roles in sensory and motor functions. The integrity of these regions is essential for overall brain function. H&E staining (**Figures 1e, f**) revealed that the hippocampal architecture in the Sham group was intact, whereas the MCAO/R group exhibited disrupted cellular organization, with a significant presence of necrotic cells (indicated by red arrows in **Figure 1f**) and tissue vacuolation due to apoptosis or edema (indicated by orange arrows in **Figure 1f**). Notably, aCD19 Ab treatment markedly improved tissue morphology and preserved cellular structural integrity in the infarcted region compared to other groups.

To further assess apoptosis in the cortex, we performed TUNEL staining. The cortical region in the Sham group maintained morphological structural integrity, with only approximately 1% of cells exhibiting positive signal, which could largely be attributed to false-positive staining. In contrast, the MCAO/R group exhibited a significant increase in TUNEL-positive cells, with approximately 61.6% of cortical cells undergoing apoptosis, indicative of substantial cell loss within the ischemic region. Remarkably, aCD19 Ab treatment significantly reduced apoptotic cell death, with only ~18% of cortical cells staining positive for TUNEL, suggesting preservation of neuronal integrity (**Figures 1g, h**). Collectively, these findings demonstrate that aCD19 Ab confers neuroprotection in the MCAO model, significantly reducing apoptosis in both the cortex and hippocampal CA region (statistical analysis presented in **Supplementary Figure S1d**).

### 3.2 aCD19 Ab treatment alleviated mortality and ameliorated functional outcomes

**Figure 2:**
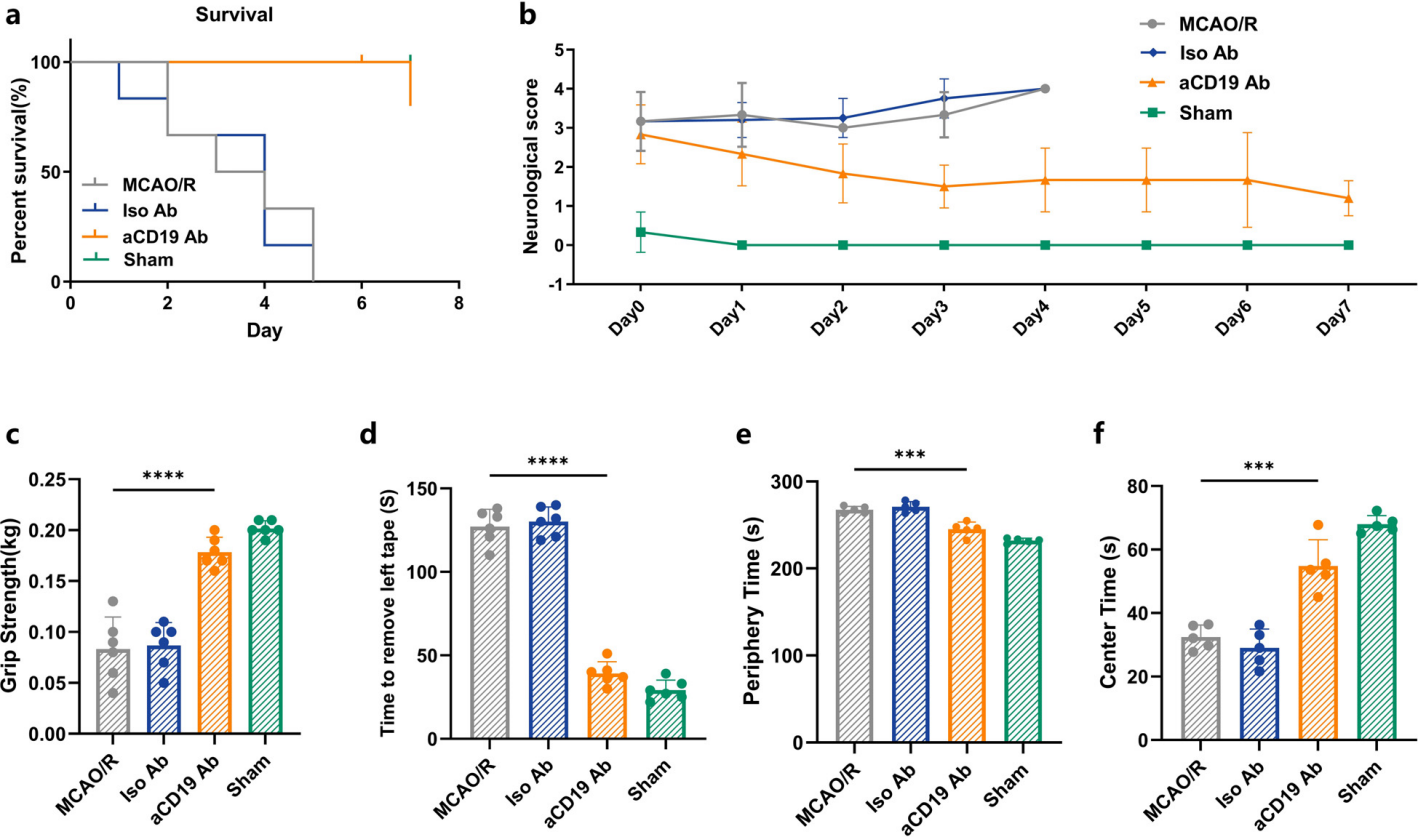
aCD19 Ab treatment alleviated mortality and ameliorated functional outcomes. **(a)** Survival rate of 7 days of MCAO/R mouse (Mantel-Cox test and Gehan-Breslow-Wilcoxon test, n=6, *p* < 0.001). **(b)** 7-day recording of scores of Zea Longa grade after MCAO/R (data were presented as mean ± SD, n=6). **(c)** Comparison of grip strength (data were presented as mean ± SD, n=6, ****p < 0.0001). **(d)** Comparison of tape moving test (data were presented as mean ± SD, n=6, ****p < 0.0001). **(e)** Open field test. Time spent in the center of the open field maze (data were presented as mean ± SD, n=5, ***p < 0.001). **(f)** Open field test. Time spent in the periphery of the open field maze (data were presented as mean ± SD, n=5, ***p < 0.001).

A series of neurological function and behavioral tests were performed to assess the sensory and motor abilities of the mice after MCAO/R. All mice underwent pre-training before the MCAO operation. Compared to the MCAO/R mice and the group that received Iso Ab administration, treatment with aCD19 Ab resulted in significantly lower stroke-induced mortality (**Figure 2a**), suggesting that aCD19 Ab improves both neurological function and endpoint outcomes post-ischemic stroke. Three commonly utilized motor behavior tests were employed to evaluate motor deficits in stroke mice. The Zea Longa (0–4) scores were used for assessing neurological outcomes in animals, where higher scores indicate more severe neurological deficits. After the MCAO operation, all mice developed a consistent level of focal nerve function loss. By day 4, the symptoms of the MCAO/R and Iso Ab groups further progressed to the level where the mice were unable to walk spontaneously. In contrast, mice administered with aCD19 Ab, though still symptomatic, showed compensation for focal neurological impairment that did not hinder normal eating and drinking. Over time, these mice exhibited a gradual and steady recovery of consciousness and walking function to normal levels (**Figure 2b**). The grip strength test was used to assess forelimb motor function. Compared to the Sham group, MCAO modeling significantly diminished the grip strength scores of the mice (higher scores indicate greater grip strength). The grip strength test was used to assess forelimb motor function. Compared to the Sham group, MCAO modeling significantly diminished the grip and weightlifting scores of the mice (higher scores indicate greater grip strength). At 48 hours post MCAO/R, although still not comparable to the Sham group and normal baseline values, the grip strength score of aCD19 Ab-treated mice was significantly higher than that of both the MCAO/R and the Iso Ab groups (**Figure 2c**). We employed the adhesive removal test to judge the sensory and motor integration capacity of the mice. During this test, the mice were required to detect and coordinate their entire body to remove a sticker placed on their left forelimb, thereby evaluating their higher-order sensory and motor abilities. The time taken by MCAO/R-induced mice to remove the sticker was significantly prolonged compared to the aCD19 Ab group (**Figure 2d**). Exploratory locomotor function and anxiety-related behavior were evaluated 3 days after the stroke using the open field test, specifically by measuring the time spent in the center of the area. We observed that both the MCAO/R and the Iso Ab groups exhibited a longer residence in the peripheral region, suggesting a lack of significant exploratory behavior. In contrast, mice treated with aCD19 Ab demonstrated an extended stay in the central region and displayed more vigorous exploratory behavior (**Figure 2e**). Detailed locomotor pathways of the mice are provided in the **Supplementary Materials** (**Supplementary Figure S2f**). These behavioral testing results revealed that neurological function showed a short-term partial recovery in the aCD19 Ab-treated mice compared to the MCAO/R group, suggesting a protective effect of the treatment.

### 3.3 Evaluation of aCD19 Ab treatment in the MCAO/R model using MRI and TEM

**Figure 3:**
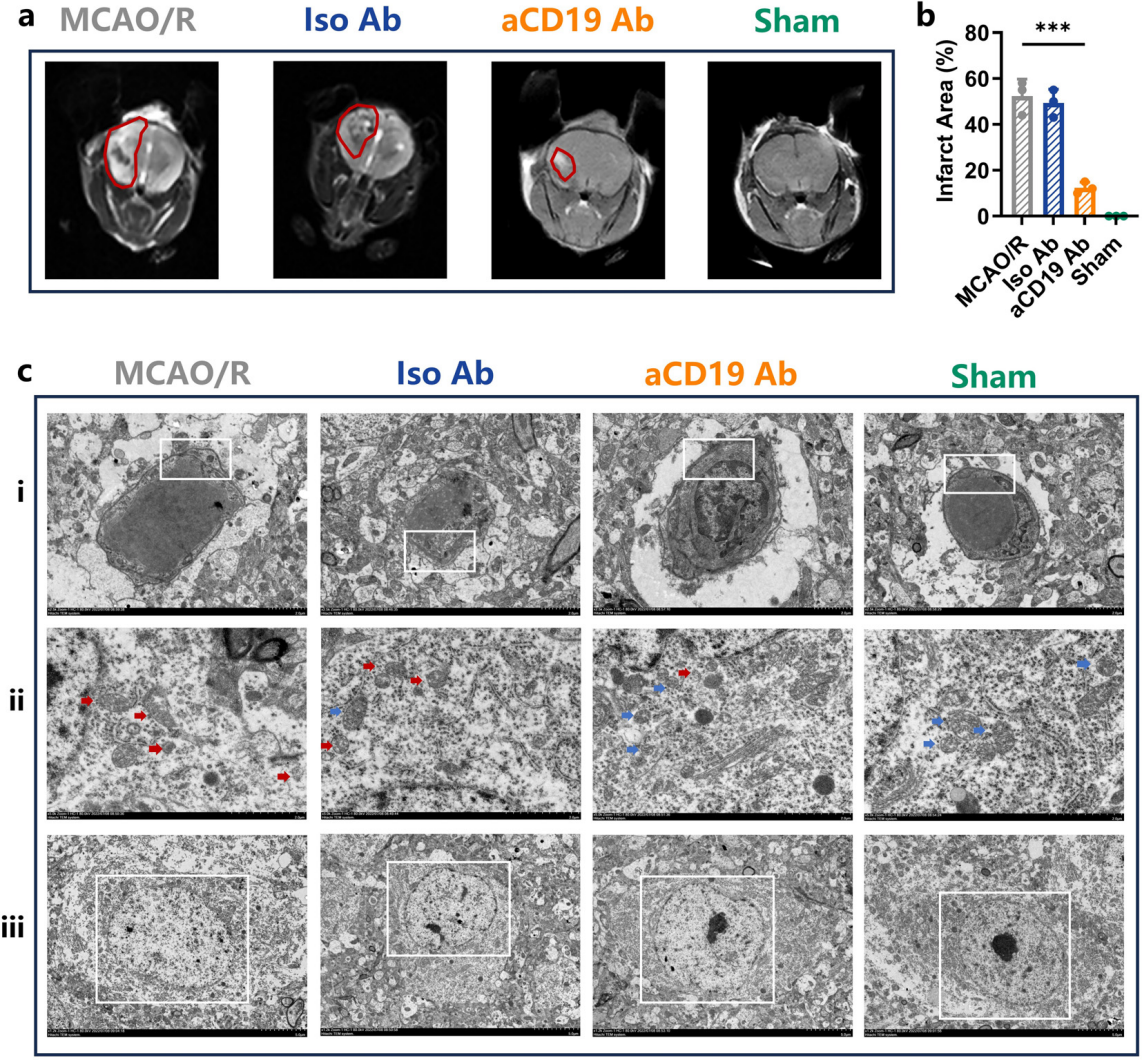
aCD19 Ab treatment reduced edema, reduced mitochondrial damage in the MCAO/R model. **(a)** Representative images showed ischemic lesion on T2wt MRI (Red Outlined). **(b)** MRI infarction area data were presented as mean ± SD, n=3, ***p < 0.001. **(c)** The TEM scanning displayed the subcellular structures of infarct area. (i) Magnification: 2.5kX – Swelling of vascular endothelial cells due to ischemia is evident, particularly in the MCAO/R group, while the aCD19 Ab-treated group exhibits less severe swelling (The White Rectangular Region). ii. Magnification: 5.0kX – Blue arrows indicate intact mitochondria, whereas red arrows highlight damaged mitochondria. iii. Magnification: 1.2kX – The MCAO/R group demonstrates nuclear apoptosis, whereas the aCD19 Ab-treated group and Sham group maintain relatively normal nuclear and nucleoplasmic integrity (The White Rectangular Region).

To further employ advanced imaging techniques in detecting substantial changes within ischemic brain regions, we performed MRI scans on the mice. The MRI images and quantitative data demonstrated that aCD19 Ab treatment significantly reduced infarct volume and cerebral edema, as indicated by a decrease in the irregular high-density signal and its peripheral extension within the red-circled region on T2-weighted MRI images, compared to the MCAO/R and Iso Ab groups. As expected, the Sham group exhibited no visible ischemic lesions (*p* < 0.05, **Figures 3a, b**).

To further investigate ultrastructural alterations in the ischemic region, we utilized transmission electron microscopy (TEM) to assess changes in the microvascular wall and alterations in the subcellular structure of neurons. In both the MCAO/R and Iso Ab groups, the vascular walls of microvessels exhibited signs of edema, accompanied by an expansion of the perivascular space. In contrast, aCD19 Ab-treated mice displayed noticeably milder vascular edema, suggesting a potential role in maintaining microvascular integrity. These pathological changes in the cerebral microvasculature are likely influenced by ischemia-reperfusion injury, pro-inflammatory cytokine activity, and oxidative stress following stroke (**Figure 3ci**, with representative changes highlighted in the white box). The observed improvements in vascular structure indicate that aCD19 Ab treatment may contribute to post-stroke angiogenesis.

Mitochondrial cristae integrity and quantity are critical for mitochondrial function, affecting cellular ATP production and water exchange pumps on the membrane surface, which in turn influence cellular edema and survival. In the Sham group, TEM revealed mitochondria with intact cristae structures in neurons (**Figure 3cii**, **Supplementary Figure S2**, indicated by blue arrows). Following the MCAO operation, there was a marked disruption and disappearance of mitochondrial cristae structures, along with morphological changes and swelling of the mitochondria in neurons (**Figure 3cii**, indicated by red arrows). In contrast, aCD19 Ab treatment effectively preserved mitochondrial integrity, maintaining cristae structure and reducing mitochondrial damage (**Figure 3cii**, indicated by blue arrows). Additional high-resolution TEM images are provided in **Supplementary Figure S2**.

Consistently, TEM findings also revealed a reduction in microvascular wall swelling and a decrease in neuronal apoptosis following aCD19 Ab treatment (**Figures 3ci, iii**). These findings, at both the imaging and subcellular structural levels, further confirm that aCD19 Ab treatment significantly mitigates stroke-induced pathological changes in brain tissue.

### 3.4 aCD19 Ab treatment facilitated reconstruction of the vascular network in ischemic stroke

**Figure 4:**
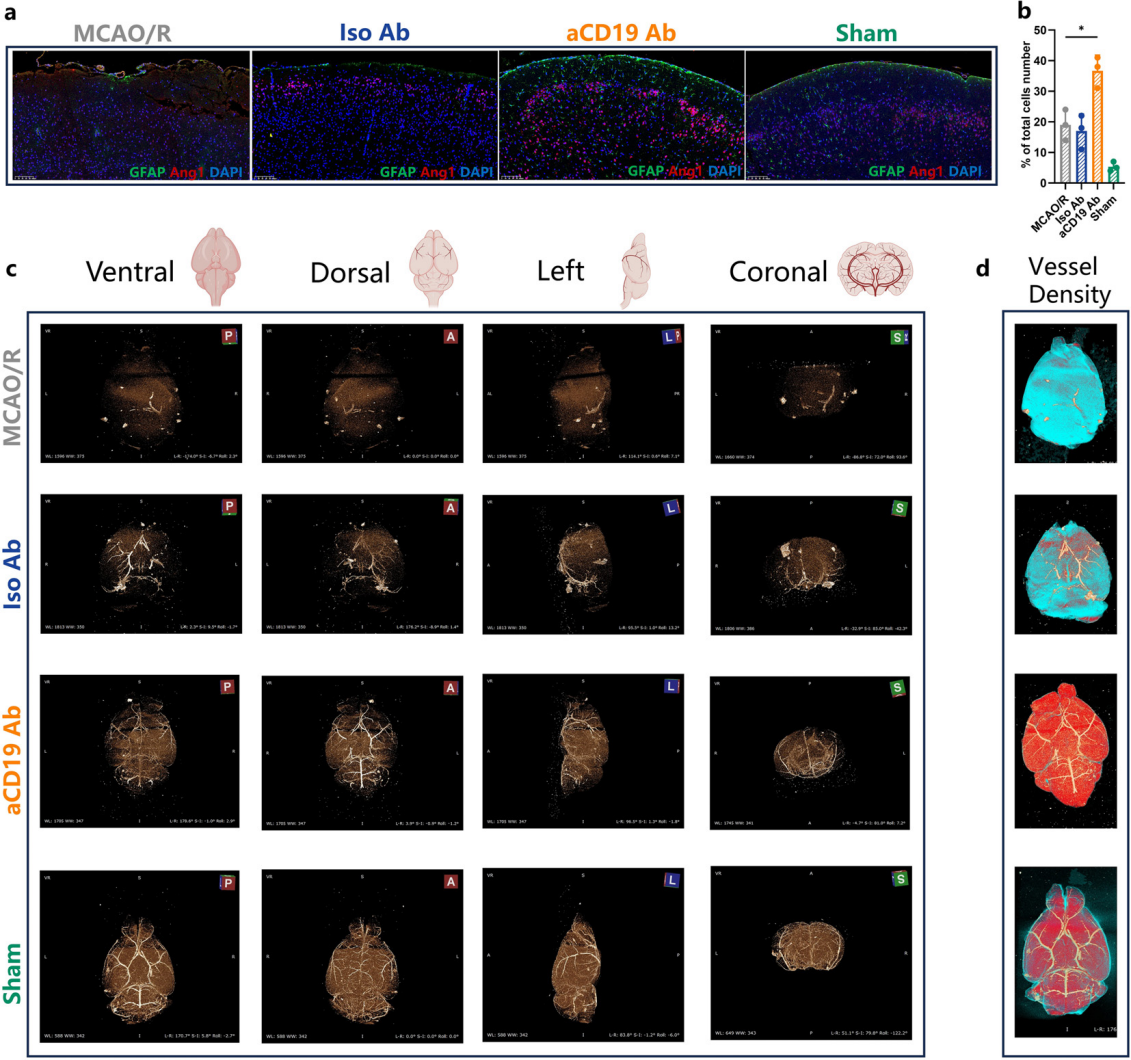
Reconstruction of vascular network in ischemic stroke. **(a)** The Angiopoietin 1 (Red) and GFAP (Green) staining exhibited the angiogenesis and the astrocytes in the cerebral tissues in 20 times magnification (Scale bar=100 μm). **(b)** The Angiopoietin 1 level of infarct area (data were presented as mean ± SD, n=3, *p < 0.05). **(c)** the 3D vascular network in ischemic stroke: Square A stood for anterior perspective; Square P stood for Rear perspective; Square L stood for the left side perspective; Square S stood for Coronal perspective. **(d)** The vessel density depended reconstruction of CT scan results: higher vessel density area (Red); lower vessel density area (Blue).

The cerebral vascular system is an intricate and highly interconnected network, with stroke research traditionally focusing on the cerebral arteries, which play a crucial role in maintaining cerebral perfusion. The fundamental structure of the cerebral vasculature is a complex network of major arteries. The vascular structures of mice and humans are similar, both based on the circle of Willis, interconnected by communicating arteries and extending into major cerebral artery trunks, which can be visualized and compared using CTA imaging. Firstly, on a microscopic level, we compared the angiogenesis-related marker Ang1. Immunofluorescence analysis revealed that Ang1 expression was significantly higher in the aCD19 Ab-treated group compared to the MCAO/R and Iso Ab groups (**Figure 4a**). The increased fluorescence intensity of Ang1 suggests that aCD19 Ab treatment exerts a protective and pro-angiogenic effect on damaged neural and vascular structures following stroke (**Figure 4a**, **Supplementary Figure S3a**). This difference was confirmed to be statistically significant (**Figure 4b**).

The compound MICROFIL was perfused through the aortic cannula to visualize the blood vessels, enabling a 3D display of the cerebral vascular network in mice from ventral, dorsal, left, and coronal perspectives using micro-CT scanning. Following MCAO induction, despite filament withdrawal, we observed persistent physical obstruction in the affected cerebral vessels. This blockage was due to vascular spasms triggered by vessel stimulation and severe cerebral edema following infarction. As a result, CT failed to reveal the blood vessels, and significant disruptions in the integrity of the Circle of Willis were noted. Additionally, the cerebral blood vessels in the contralateral hemisphere were similarly affected and not visible on imaging. Following treatment with aCD19 Ab, although the middle cerebral artery directly impacted by the filament occlusion still exhibited irreversible vasospasm and the blood vessels in the ischemic hemisphere remained notably diminished, vessels in other regions—particularly the contralateral hemisphere—appeared relatively preserved. These findings suggest that aCD19 Ab intervention reduced vascular spasm and spontaneous occlusion throughout the brain, thereby maximizing the preservation of the cerebral vascular network’s integrity (**Figure 4c**). Based on CT scan data, we reconstructed an overall brain image using a heat map-like expression to analyze blood vessel density, representing the degree of blood supply. We speculated that the blue regions, with less cerebral vascular density, had lower blood flow, while the red regions, with higher cerebral vessel density, had increased blood flow. The aCD19 Ab group exhibited significantly higher blood vessel density compared to both the MCAO/R and Iso Ab groups (**Figure 4d**). These findings visually demonstrated changes in cerebral blood supply and suggested a recovery of the angiogenic response following treatment, highlighting the potential implications for aCD19 Ab therapy strategies targeting stroke-induced angiogenesis restoration.

### 3.5 Regulation of cerebral and systemic immune response by anti-CD19 Ab treatment

**Figure 5:**
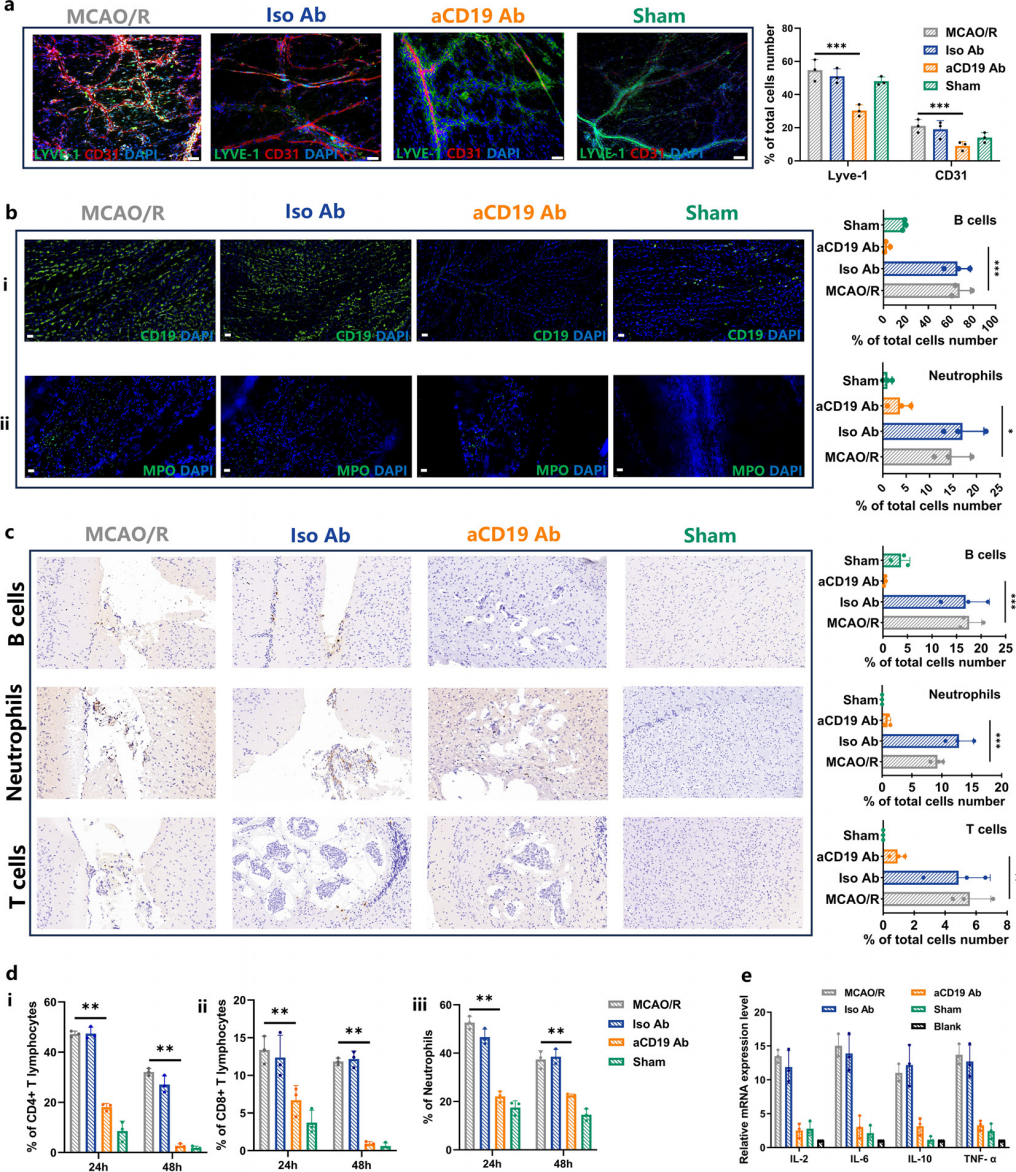
Injection of aCD19 Ab reduced meningeal local and systemic inflammatory response. **(a)** The IF co-staining results of LYVE-1 and CD31 at 20 times magnification (Scale bar=100 μm) (data were presented as mean ± SD, n=3, ***p < 0.001). **(b)** (i) The IF staining results of CD19 in meninges tissue at 60 times magnification (Scale bar=20 μm) (data were presented as mean ± SD, n=3, ***p < 0.001). ii. The IF staining results of MPO in meninges tissue at 60 times magnification (Scale bar=20 μm) (n=3, *p < 0.05). **(c)** Representative IHC staining of B cells, Neutrophils and T cells in the infarct area at 60 times magnification (Scale bar=20 μm) and their statistical analysis of the ratio of the positive cells to the total cells number (data were presented as mean ± SD, n=3, ***p < 0.001 and **p < 0.01). **(d)** The Flow Cytometry results of immune cells in peripheral blood of MCAO/R mouse. (i) CD4^+^T cells. ii. CD8^+^T cells. iii. Neutrophils (data were presented as mean ± SD, n=3, **p < 0.01). **(e)** Expression of the inflammatory cytokines in cerebral parenchyma (data were presented as mean ± SD, n=3).

Recent studies have found that MLVs are widely distributed throughout the central nervous system, including the sieve plate, dorsolateral meninges, base of the brain, and around the spinal cord. These vessels play a crucial role in storing, migrating, and transporting immune cells within the cerebral immune system, as well as connecting the central and peripheral immune systems. Additionally, MLVs are involved in the removal of brain macromolecules and in mediating immune responses. Impaired clearance of factors may lead to edema and cerebral immune dysfunction, further exacerbating post-stroke inflammation. Given this, we sought to investigate the interaction between the meningeal immune cells induced by aCD19 Ab therapy after stroke. The specific markers CD31 and LYVE-1 antibodies were employed for distinguishing between blood vessels and lymphatic vessels (**Figure 5a**). **Supplementary Figure S3b** presents the whole-mount meningeal staining, with the region of interest (ROI) marked by a white box. This designated ROI is located on the infarcted side of the brain following MCAO. The images displayed in **Figure 5a** were specifically selected from this ROI, ensuring a consistent and representative assessment of meningeal lymphatic and vascular changes in response to aCD19 Ab treatment. Double-staining with LYVE-1 and CD31 markers confirmed that lymphatic endothelial proliferation in the meningeal area of mice was increased in the MCAO/R and Iso Ab groups, while it was relatively lower in the aCD19 Ab group (**Figure 5a**). Individual fluorescence channel images are displayed separately in **Supplementary Figure S3c**.

Interestingly, the infiltration of B cells and neutrophils into the meningeal lymphatic vessels of MCAO/R mice was significantly higher than that observed in aCD19 Ab-treated mice (**Figure 5b**). To verify the consistency of immune cell subpopulation changes between the central nervous system and the peripheral system, we further investigated the number of immune cells in both the brain parenchyma and peripheral blood. Flow cytometry results revealed that the proportion of B cells in the brain parenchyma was significantly higher in the MCAO/R group compared to the Sham group, whereas aCD19 Ab treatment effectively reversed this increase (**Supplementary Figure S4a**).

Moreover, as previous studies have demonstrated cross-regulation among immune cell subtypes, we also assessed T-cell and myeloid cell populations within the brain (**Supplementary Figures S4b, c**). IHC analysis provided a visual representation of these findings, demonstrating that the recruitment of T cells, B cells, and neutrophils in the brain parenchyma was significantly elevated in the MCAO/R group compared with the Sham group. However, aCD19 Ab treatment effectively reduced immune cell infiltration and migration (**Figure 5c**).

These central immune-modulatory effects were further corroborated by peripheral blood analysis, where aCD19 Ab treatment resulted in a significant reduction in CD4^+^ T lymphocytes, CD8^+^ T lymphocytes, and neutrophils as early as 24 hours post-treatment, with this effect persisting up to 48 hours (**Figure 5d**, flow cytometry data shown in **Supplementary Figure S4d**). Meanwhile, cytokines play an important chemotactic role in the immune system. Using qPCR technology, we detected cytokine expression and found a significant increase in levels of *Il-2*, I*l-6*, *Il-10*, and *Tnf-α* within the MCAO/R group. However, treatment with aCD19 Ab effectively reversed this elevation (**Figure 5e**). Taken together, these results suggest that aCD19 Ab treatment modulated local and systemic immune infiltration and cytokine expression after ischemic stroke.

### 3.6 Meningeal immune cell infiltration and peripheral immune system changes in stroke patients

**Figure 6:**
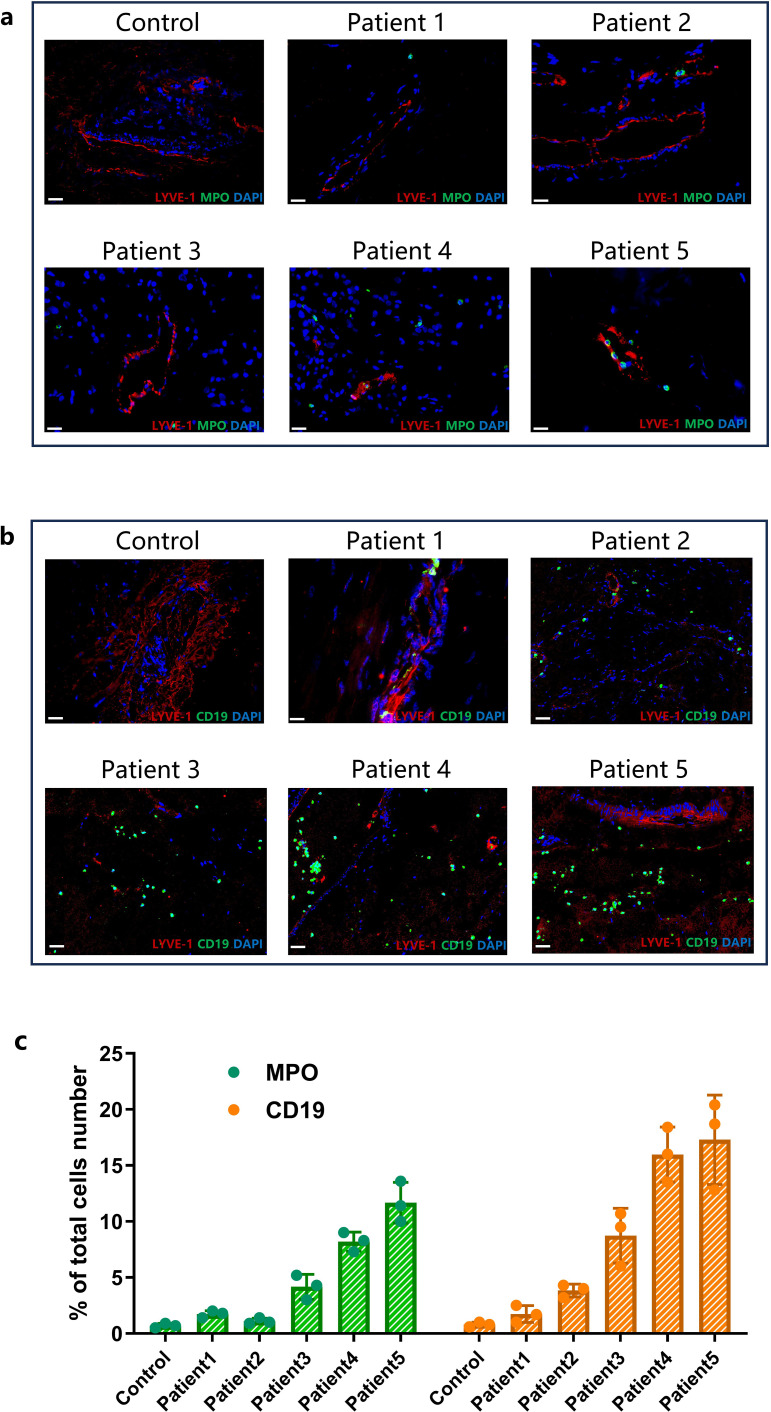
The status of meningeal immune cell infiltration and peripheral immune cell changes in stroke patients. **(a)** The IF co-staining results of LYVE-1 (Red) and MPO (Green) in meningeal tissue at 60 times magnification (Scale bar=20 μm). **(b)** The IF co-staining results of LYVE-1 (Red) and CD19 (Green) in meningeal tissue at 60 times magnification (Scale bar=20 μm). **(c)** Comparison of the ratio of the CD19 and MPO positive cells to the total cells number in meningeal(data were presented as mean ± SD, n=3).

Meningeal tissues were collected from five stroke patients who underwent decompressive craniectomy (patient details provided in **Table 1**; PRISMA flowchart shown in **Supplementary Figure S6**). Immunofluorescence staining using LYVE-1, MPO, and CD19 was performed to observe meningeal immune cell infiltration (individual fluorescence channel images available in **Supplementary Figure S5**). The expression levels of MPO and CD19 varied among patients, suggesting differences in neutrophils and B cells infiltration in meningeal tissues (**Figures 6a, b**). both MPO and CD19 expression in the meningeal tissue exhibited a consistent upward trend among patients, suggesting a potential correlation between immune cell infiltration and stroke severity (**Figure 6c**). Furthermore, peripheral blood lymphocyte counts and cytokine profiles revealed trends that were consistent with the infiltration patterns observed in the meninges. Specifically, patients with more severe clinical conditions had higher MPO and CD19 counts, with both markers showing a parallel increase in expression (**Table 1**).

Due to the small sample size, we were unable to conduct a large-scale cohort study or a controlled trial. Within this limited cohort, we identified a potentially indicative trend: increasing MPO and CD19 expression correlated with greater disease severity. The patients’ status and outcomes, evaluated using GCS, mRS, NIHSS, and MBI scores, all showed a negative correlation with the patients’ peripheral B cell and neutrophil counts and cytokine levels, particularly the expression of IL-2 and IL-6 (**Table 1**).

## 4 Discussion

This comprehensive study employed a multifaceted analytical approach to assess the effects of B cell inhibition in an ischemic stroke model. The macroscopic imaging techniques, such as TTC and MRI, revealed a significant reduction in infarct size and volume, indicating the effectiveness of B cell inhibition. Further corroborated by histological examinations like H&E staining and TUNEL assays, these findings demonstrated improved microstructural integrity and reduced apoptosis in areas treated with the aCD19 antibody.

Meanwhile, the study highlighted the anti-inflammatory benefits of CD19^+^ B cell depletion. By moderating the activation of inflammatory factors and reducing cytokine levels, this approach effectively decreased the recruitment of chemotactic immune cells. This modulation of the immune response also negated the need for a rise in anti-inflammatory cytokines, explaining the observed reduction in IL-10 levels in the aCD19 Ab group.

B cells are critical in host defense by the secretion of antibodies as well as by generation of chemokines/cytokines and antigen-presenting to T cells, thereby influencing the activation of T cells and shaping the overall inflammatory environment (31). B cells can also influence myeloid cell behavior, particularly by modulating macrophage polarization via cytokine production or direct cell-cell interactions (32, 33). In our study, while we acknowledge that B cells are not the most abundant immune cells infiltrating the stroke site—especially when compared to myeloid cells—the observed effects of B-cell depletion are likely a result of multifactorial interactions, involving both direct and indirect immunomodulatory effects rather than the suppression of a single immune cell type in isolation. PCOS is a chronic inflammatory disease. Our previous findings also revealed that CD19+ B cell-depleted mice were resistant to DHEA-induced PCOS, while aCD19 Ab treatment markedly ameliorated pathological phenotypes in PCOS mice by inhibiting local and systemic inflammation (25, 34). Thus, we hypothesize that B-cell depletion disrupts the overall inflammatory cascade by reducing the production of pro-inflammatory cytokines and modulating interactions between myeloid cells and T cells. The disruption of specific inflammatory signaling branches within this cascade may ultimately contribute to the observed protective effects in stroke outcomes.

Vascular occlusion resulting from MCAO, coupled with a local inflammatory response leading to brain edema, may result in physical compression, vasospasm, micro-vascular thrombosis, and subsequent vascular occlusion. Based on CTA data, the density and integrity of cerebrovascular network (**Figure 4c**) showed that B cell inhibition facilitated the development of compensatory collateral circulation, likely due to the amelioration of cerebral edema and prevention of further vascular damage. Anti-CD19 treatment enhanced the regeneration of microcirculation and promoted cerebral blood flow reconstruction, thereby improving perfusion to unaffected brain territories. TEM technology revealed less severe microvascular wall edema in the aCD19 Ab group compared to the control group (**Figure 3ci**). The expression of the angiogenesis-related marker Ang1 was significantly higher in the aCD19 Ab group, indicating reactive angiogenesis under ischemic and hypoxic stimulation. This compensatory vascular proliferation may improve the blood supply in the peri-ischemic region (penumbra). Specifically, the restructured cerebral blood flow, as seen in TEM results, showed alleviated mitochondrial damage and neuronal structural damage (**Figures 3cii, iii**). The disruption of mitochondrial cristae plays a direct role in the development of edema through a well-established mechanistic cascade rooted in cellular bioenergetics and ionic homeostasis: Cristae house the electron transport chain (ETC), which is essential for oxidative phosphorylation and ATP synthesis. Structural damage to these cristae severely impairs ETC efficiency, leading to ATP depletion.

Given that neurons rely heavily on ATP to fuel Na^+^/K^+^-ATPase pumps, which maintain ionic homeostasis by extruding intracellular Na^+^, ATP depletion results in pump failure and subsequent Na^+^ accumulation within the cytoplasm (35). This intracellular sodium overload creates an osmotic gradient that drives water influx, ultimately causing neuronal swelling and cytotoxic edema. This mechanism is consistent with previous findings demonstrating that cristae disruption exacerbates post-ischemic edema (36).

Moreover, mitochondrial cristae damage promotes the opening of the mitochondrial permeability transition pore (mPTP), further destabilizing membrane potential and enhancing the production of reactive oxygen species (ROS) (37). The interplay between oxidative stress and ionic imbalance likely amplifies edema severity, highlighting the critical role of mitochondrial integrity in post-ischemic brain injury.

The vessel density color map (**Figure 4d**) indicated that regions with higher cerebrovascular density (depicted in red) have a better blood supply, especially in the intact contralateral hemisphere, while areas with lower cerebrovascular density (depicted in blue) suggested reduced blood supply. This micro-CT data interpretation may provide a novel method to assess changes in cerebral blood flow. The insertion of foreign objects impacts the vascular endothelium, and the MCAO procedure itself causes physical damage to the structure of cerebral blood vessels (38, 39). Consequently, the affected vessels may undergo permanent occlusion (40). Admittedly, this anti-CD19 immunomodulatory treatment cannot fully restore the ischemic core brain tissue but does prevent further expansion of the infarct area, explaining why the infarct area was not completely eliminated, as shown in the TTC and MRI results (**Figure 1**). Moreover, measurements of brain water content indicate that MCAO mice had severe cerebral edema before treatment. Clinical observations suggest that cerebral edema can lead to brain herniation, resulting in physical compression and closure of blood vessels (41). This is directly evidenced by the reduced cerebral vasculature observed in the MCAO/R group in the micro-CT images.

When a stroke occurs, the body’s response includes a surge in immune cells, inflammatory cytokines, and ROS due to ischemia-reperfusion. These elements act as “antigens” that stimulate a systemic immune response, allowing immune cells to migrate into the brain through a disrupted BBB (19). Current research is keenly focused on understanding how these peripheral immune cells traverse into the CNS, their roles in the pathology of stroke, and their potential as therapeutic targets (42).

An important component of this immune interaction involves the MLVs, which are located within the dura mater. These vessels serve crucial functions, including the drainage of interstitial fluid, macromolecules, and immune cells from the cranial cavity, regulating the immune responses within the brain (16, 43, 44). The role of MLVs has drawn significant attention, hypothesizing that alterations in this system or changes in the local immune microenvironment might reciprocally influence each other, potentially affecting the progression and outcome of stroke (45, 46). In ischemic region, MLVs are implicated in penetrating the injured brain parenchyma. This invasion assists in reducing brain edema by providing a pathway for the drainage of cerebral interstitial fluid and by promoting angiogenesis (47). Such multifaceted roles underscore the critical importance of the meningeal lymphatic system following a cerebral infarction and point to its potential as a therapeutic target for managing ischemic stroke.

To cross-validate the results obtained from animal models with clinical reality, it is crucial to have access to human samples that accurately represent dynamic cerebral immune changes. Due to ethical and surgical constraints, direct access to brain tissues from stroke patients during treatment is restricted, and postmortem samples often fail to capture the rapid immune system changes occurring during stroke onset and progression. Therefore, in this study, studying real-time dynamic cerebral immune changes relies on alternative sources like dura mater samples obtained during decompressive craniectomy surgeries. These samples, which are typically discarded as medical waste, provide a unique opportunity for examining the inflammatory responses and immune cell activities in the context of stroke. According to the analysis of meningeal samples from MCAO mice, stroke-induced inflammatory responses or immune cells migration can increase LYVE-1 expression and CD31 endothelial interaction, which was considered an interplay between terminal lymphatic vessels and capillaries. Moreover, studies have demonstrated that preconditioned anti-CD19 treatment in MCAO mouse models substantially reduces B cell presence and affects the infiltration of neutrophils, mirroring observations in human samples (**Figure 6a**). In the immunofluorescent staining of dura mater from stroke patients, there was a positive correlation between the expression of MPO marker and CD19 marker (**Figure 6c**). This indicates a positive correlation between the number of neutrophils and B cells within the MLVs. But the initiating factors and specific mechanisms underlying this relationship remain unclear and warrant further investigation. The variation in immune cell infiltration in MLVs observed in this small-sample study was negatively correlated with patient prognosis. Additionally, the increase in lymph vessel density and diameter of MCAO mice may be attributed to abnormal intracranial pressure caused by edema (**Figure 5a**). As lymphatic drainage is essential for removing excess fluid from the parenchyma, the changes diameter and density of MLVs poststroke result in a greater outflow rate of drainage under high-pressure conditions (48). This suggests that when edema results in higher intracranial pressure after stroke, meningeal lymphatic drainage is activated, decreasing water content (**Figure 1d**) and alleviating edema (49). These insights contribute to understanding the intricate interplay among the immune system, cerebral edema, and the MLVs in the context of stroke.

Pre-treatment studies using stroke animal models are a common research approach (50). Although clinical interventions predominantly focus on post-stroke treatments, the severe consequences of stroke and the limitations of current therapies in fully restoring neurological function highlight the urgent need for effective preventive strategies in high-risk populations. This necessity parallels the use of antiplatelet and anticoagulant therapies in conditions like hypertension to avert severe events (51–53). Notably, numerous immunomodulatory and immunosuppressive drugs developed for other diseases already exist (54, 55). Leveraging the principles and mechanisms underlying these therapies, we can investigate preventive immunotherapeutic methods specifically for stroke. Immunomodulatory treatments generally require time to become effective; thus, administering them after stroke onset may not yield timely benefits compatible with the acute nature of stroke progression. This discrepancy underscores the potential value of exploring reversible, non-permanent pharmacological pre-treatments administered before stroke occurrence in high-risk individuals—a strategy that could become viable in the future.

Such preventive pre-treatment strategies could proactively modulate the immune system, thereby reducing the risk of stroke or mitigating its severity upon occurrence. Additionally, this approach avoids the long-term side effects and physiological burdens associated with prolonged use of immunosuppressants. Appropriately adjusting the immune response in high-risk populations also offers a new avenue for personalized medicine.

Short-term prophylactic B cell inhibition aligns with studies suggesting that B cell involvement is either negligible in the acute phase or beneficial during chronic stages of stroke. This concept of aCD19 Ab treatment aims to reduce the early exaggerated inflammatory response induced neural damage, presenting a viable therapeutic approach. In translating aCD19 Ab treatment into a viable therapeutic approach, several challenges must be considered, including the heterogeneity of human stroke pathology, which is highly variable and often influenced by comorbidities such as hypertension, diabetes, and atherosclerosis. These conditions can significantly alter immune responses and impact treatment efficacy, making it difficult to directly translate findings from controlled preclinical models to clinical applications. Additionally, there are fundamental differences in B-cell subset composition, activation pathways, and regulatory mechanisms between murine and human immune systems, further complicating the extrapolation of experimental results. Another critical factor is the optimization of dosing regimens, as achieving a balance between immune suppression and neuroprotection is essential. While aCD19 Ab treatment presents a promising immunomodulatory strategy, excessive suppression may increase the risk of infections, whereas insufficient modulation may fail to provide therapeutic benefits. Addressing these challenges is imperative to refine and enhance the clinical applicability of aCD19 Ab treatment in stroke therapy. Future studies should focus on long-term safety, personalized treatment considerations, and the broader immunological implications to ensure both efficacy and safety in diverse patient populations.

Nevertheless, several questions need to be addressed. Firstly, while our study focused on the benefits of aCD19 Ab treatment in a stroke model, some reports indicate that long-term outcomes may differ (56). The complex and dynamic process of neural repair and regeneration over extended periods necessitates future studies to explore how aCD19 Ab treatment might influence long-term neurorehabilitation and functional recovery (57, 58). Secondly, our study is based on observational data from a limited cohort of stroke patients, so the generalizability of our findings to broader populations requires further investigation. The impact of different stroke subtypes, such as ischemic or hemorrhagic strokes, on the observed effects remains unclear. Further research is needed to verify the robustness of our results and to determine whether other factors, such as genetic predispositions or comorbidities, may also influence the outcomes. Thirdly, recent studies have suggested that the interplay between the immune system and the MLVs could be crucial in stroke pathogenesis (59). While our investigation touched upon the role of the MLVs, the broader dynamics of this interaction and its potential contribution to stroke-related complications are not fully understood. Future research should delve into the intricate details of how MLVs may influence immune responses in the context of stroke, providing a more comprehensive understanding of the mechanisms involved.

## 5 Conclusions

In summary, our study offers insights into the potential benefits of the CD19^+^ B cell depletion in stroke management. We observed consistent results in meningeal lymphatic vessels across both clinical stroke samples and animal experiments, underscoring the importance of integrating clinical and basic research. The findings suggest that short-term B-cell inhibition can alleviate early inflammatory responses and reduce neural damage caused by oxidative stress, indicating a promising therapeutic approach.

This research opens avenues for exploring immunotherapy strategies aimed at mitigating the severity of stroke. However, the study has limitations, and further research is necessary to validate the feasibility and safety of this approach. Future studies should focus on the long-term effects of B-cell inhibition on stroke recovery, the influence of genetic predispositions and comorbidities, and the detailed mechanisms by which meningeal lymphatic vessels influence immune responses in stroke. Addressing these areas will pave the way for more targeted and effective therapeutic strategies in stroke treatment.

## Data Availability

The data generated in this study are available within the article and its **Supplementary Data Files**. A structured repository of data does not exist. Additional data (e.g., deidentified raw data) are available upon request to the corresponding author, YZ, subject to necessary authorization from the Data Review Committee of the author’s institution. All data requests must comply with the Personal Information Protection Act to ensure the privacy and confidentiality of patient data. The corresponding author, YZ, will provide guidance on the privacy-preserving procedures required for data access. Requests to access the datasets should be directed to YZ, 507766@csu.edu.cn.
