# Supplementary figures and images for: CD19+ B cell depletion: a novel strategy to alleviate ischemic stroke damage
Source: Front Immunol. 2025 Apr 17;16:1528471. doi: 10.3389/fimmu.2025.1528471 (PMC12043492; doi:10.3389/fimmu.2025.1528471)

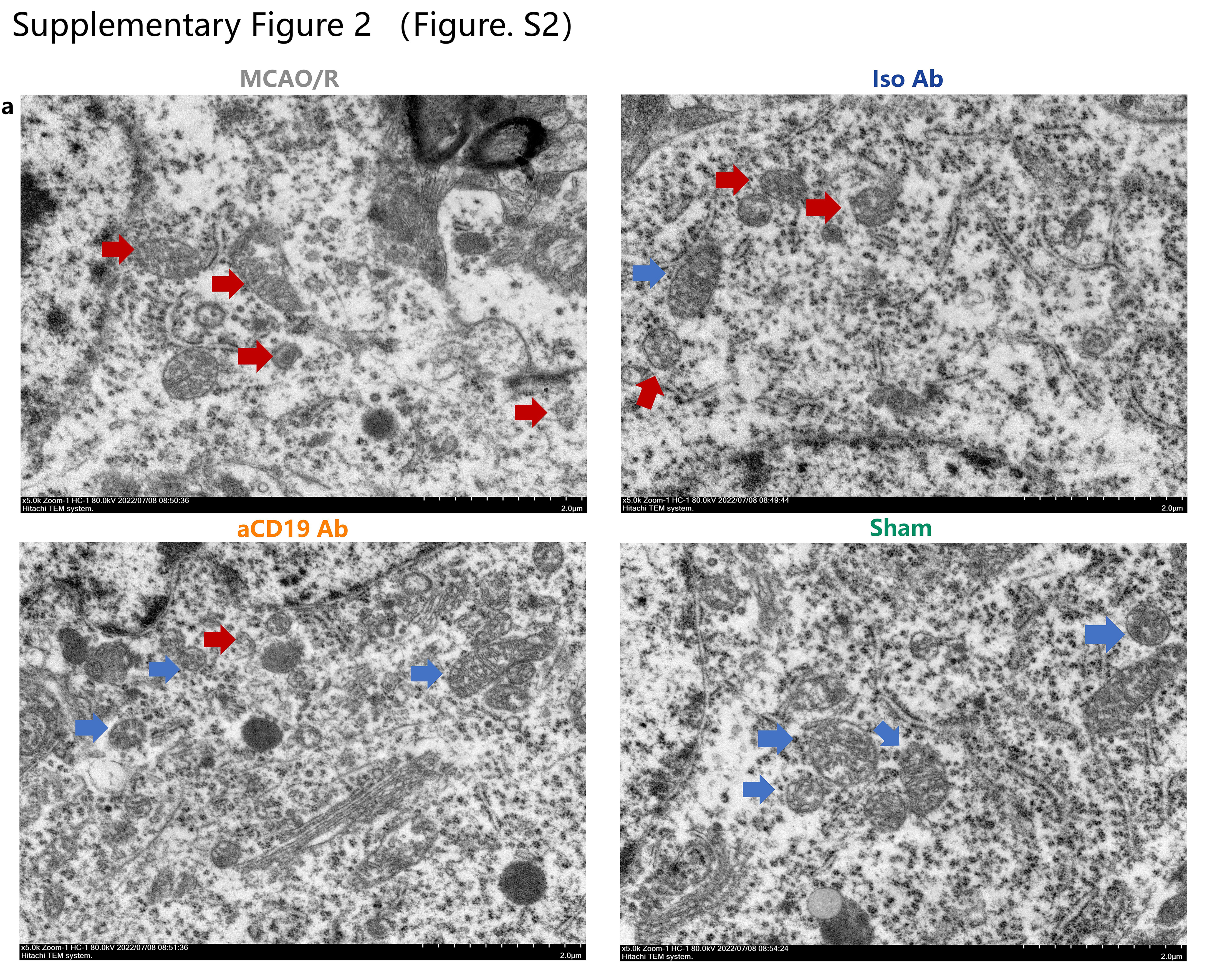

Supplement: Supplementary file 2 [file Image2.jpeg]

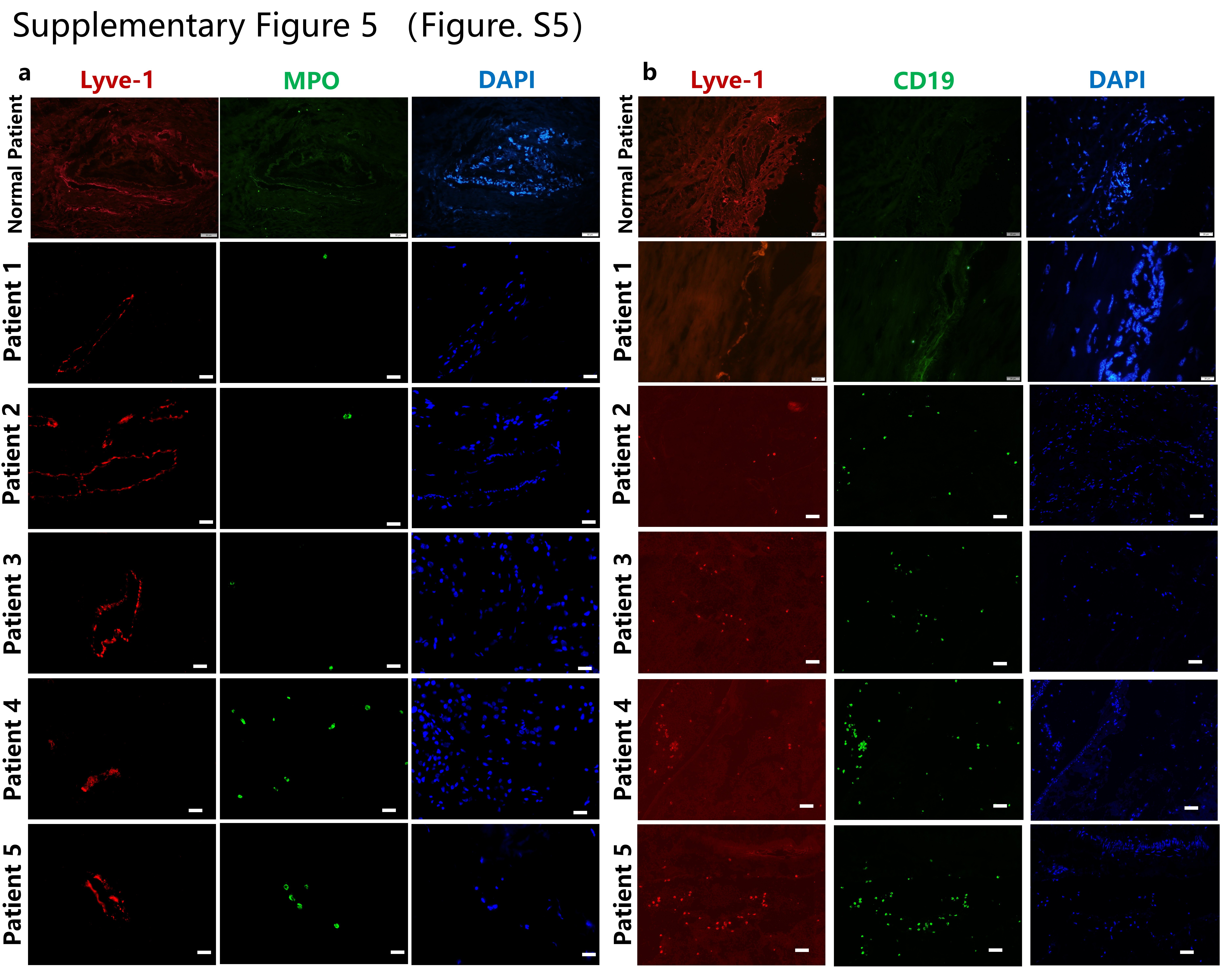

Supplement: Supplementary file 5 [file Image5.jpeg]
