# Supplementary material for: CD19+ B cell depletion: a novel strategy to alleviate ischemic stroke damage
Source: Front Immunol. 2025 Apr 17;16:1528471. doi: 10.3389/fimmu.2025.1528471 (PMC12043492; doi:10.3389/fimmu.2025.1528471)
Supplement: Supplementary file 7 [file DataSheet1.pdf]

Supplementary Material

1. Supplementary Figures

Supplementary Figure 1 (**a**) Flow cytometry analysis demonstrated the depletion of peripheral circulating B cells following intraperitoneal injection of aCD19 Ab. (**b**) Baseline levels of peripheral blood B cells in untreated mice (control group), as well as B cell proportions in the Iso Ab group and aCD19 Ab group without MCAO induction (gate on $CD45^+$). (**c**) The detailed experimental timeline and specific procedural details. (**d**) Quantification of TUNEL-positive cells, corresponding immunofluorescence images were shown in Figure 1 in the main text (data were presented as mean ± SD, n=3, ****p* < 0.001). (**e**) Image of the Grip Strength Test apparatus, which offered multiple output data modes. (**f**) Path trajectory map from the Open Field Test, depicting movement patterns of mice.

Supplementary Figure 2 High-magnification TEM images, allowing for clearer identification of labeled arrows without obstructing critical structural details. Blue arrows indicate intact mitochondria, whereas red arrows highlight damaged mitochondria.

Supplementary Figure 3 (**a**) Fluorescence images depicting GFAP (Green) and Ang1 (Red) double staining in mice from the four experimental groups. Individual channels were shown separately prior to merging (Scale bar=100 μm). (**b**) Whole-mount meningeal preparation of a mouse at 1.5 times fluorescence microscopy magnification. The white box highlighted the approximate region selected for detailed meningeal analysis, corresponding to the stroke-affected hemisphere, as shown in the main text. (**c**) Fluorescence images depicting Lyve-1 (Green) and CD31 (Red) co-staining in mice from the four experimental groups. Individual channels were shown separately prior to merging (Scale bar=100 μm).

Supplementary Figure 4 Changes in intracerebral immune cell subpopulations in MCAO mice, presented with original flow cytometry data. (**a**) Flow cytometry analysis and quantification of $CD19^+$ B cells in the four experimental groups (gate on $CD45^+$ in brain) (data were presented as mean ± SD, n=3, ****p* < 0.001). (**b**) Flow cytometry analysis and quantification of $CD3^+$ T cells in the four experimental groups (gate on $CD45^+$ in brain) (data were presented as mean ± SD, n=3, **p* < 0.05). (**c**)

Flow cytometry analysis and quantification of $CD11b^+$ myeloid cells in the four experimental groups (gate on $CD45^+$ in brain) (data were presented as mean ± SD, n=3, ***p* < 0.01). (**d**) The proportion of $CD4^+$ T cells, $CD8^+$ T cells and $CD11b^+$ $Ly6G^+$ neutrophils in the four experimental groups (gate on $CD45^+$ in brain).

Supplementary Figure 5 (**a**) Fluorescence images depicting Lyve-1 (Red) and MPO (Green) double staining in patients from the meningeal tissue. Individual channels were shown separately prior to merging (Scale bar=20 μm). (**b**) Fluorescence images depicting Lyve-1 (Red) and CD19 (Green) double staining in patients from the meningeal tissue. Individual channels were shown separately prior to merging (Scale bar=20 μm).

Supplementary Figure 6 The PRISMA chart of the clinical study.

2. Supplementary Table

Table 1. Abbreviations

aCD19 Ab	Anti-CD19 antibody
TEM	Transmission electron microscope
CTA	Computed Tomography Angiography
IL	Interleukin
TNF-α	Tumor necrosis factor-alpha
tPA	Tissue plasminogen activator
CNS	Central nervous system
MCAO/R	Middle cerebral artery occlusion/reperfusion
MLVs	Meningeal Lymphatic Vessels
BBB	Blood Brain Barrier

PBs	plasmablasts
PCs	plasma cells
PCOS	polycystic ovary syndrome
DPBS	Dulbecco's Phosphate-Buffered Saline
tMCAO	Transient middle cerebral artery occlusion
PFA	Paraformaldehyde
GCS	The Glasgow Coma Scale
mRS	The Modified Rankin Scale
NIHSS	The National Institutes of Health Stroke Scale
MBI	The Barthel Index
TTC	2,3,5-triphenyltetrazolium chloride
H&E	Hematoxylin and eosin
IF	Immunofluorescence
IHC	immunohistochemical
MPO	Myeloperoxidase
DAB	Diaminobenzidine
IOD	Integrated optical density
MRI	Magnetic resonance imaging
3D	Three-dimensional
FSE	Fast spin echo sequence
TE	Echo time

TR	Repetition time
BW	Bandwidth
SD	standard deviation
ANOVA	analysis of variance
ROI	region of interest
ETC	electron transport chain
mPTP	mitochondrial permeability transition pore
ROS	reactive oxygen species
NSFC	Natural Science Foundation of China
